# The impact of stress on depression, ill health and coping in family members caring for patients with acquired brain injury

**DOI:** 10.4102/safp.v62i1.5149

**Published:** 2020-10-08

**Authors:** Janet Walker, Lourens Schlebusch, Bernhard Gaede

**Affiliations:** 1 Department of Family Medicine, College of Health Sciences, University of KwaZulu-Natal, Durban, South Africa; 2 Department of Behavioural Medicine, College of Health Sciences, University of KwaZulu-Natal, Durban, South Africa

**Keywords:** acquired brain injury, stress, depression, ill health, caregiver burden, coping

## Abstract

**Background:**

This study investigated the impact of stress on levels of depression and ill health as an indication of psychological coping. The research sample consisted of 80 family caregivers (who are members of Headway Gauteng, located in Johannesburg, South Africa) of patients with acquired brain injury.

**Methods:**

A mixed method design of data collection was utilised that included self-report procedures (structured questionnaires and interviews) and post-interview content analyses. In addition, two individually administered measures that have been widely used in clinical practice and research were administered (a stress symptom checklist and the Beck Depression Inventory).

**Results:**

The majority of the research participants experienced high levels of stress along with an inordinate physical and mental health impact indicating that they were not able to cope up with the ongoing chronic stress of caregiving.

**Conclusion:**

Findings provide compelling evidence of the value of psychological screening for elevated stress and poor coping in family members caring for a patient with acquired brain injury in a resource-limited healthcare society. We recommend a collaborative effort between medical and psychological health practitioners in order to ensure a holistic and inclusive approach towards treatment procedures and interventions to improve coping skills in family members caring for a patient with acquired brain injury.

## Introduction

A leading cause of disability, globally and in South Africa, is brain injury and because of often-limited availability of rehabilitation and support resources, these patients can become a family member’s responsibility.^1,2,3,4,5^ Both patients with acquired brain injury (ABI) and the family members who care for them can experience very high levels of stress which can undermine their psychological and physiological well-being and thereby reduce the family member’s ability to cope with the demands associated with being an effective caregiver. This is because chronic stress can lead to serious physiological and psychological^6,7,8,9,10^ health complications and can reduce psychological resilience and adaptation.^7^ Allostasis is the body’s way of adapting to the stress response. Prolonged and unmanaged stress can disrupt this neurobiological process by producing harmful allostatic load or overload (AL).^7,8,11^ This AL can, over time, result in the breakdown of allostasis through the cumulative erosion of neurophysiological mechanisms, potentially undermining mood, cognition, organ, neuroendocrine, autonomic, immune, metabolic and other functions.^7,8,12^ Because of multiple demands made on family members caring for a patient with ABI they may be particularly susceptible to AL, which may result in adverse psychophysiological complications. For example, a study of individuals who have a gene variant involved in moderating the neurotransmitter serotonin showed that those who experienced several stressful events had a markedly higher risk of developing major depression.^13^ Furthermore, stress and the resultant AL has been suggested to be a significant contributor to the development of cardiovascular disease.^7^

The neuropsychological and other ramifications in patients with ABI often require the family caregivers to accommodate within the family system a person displaying changes in personality, behaviour and cognitive ability, amongst others.^1^ This could further increase their exposure to severe stress as they attempt to adapt to what has been referred to as the caregiver burden.^14^ Caregiver burden is a cause of significant difficulty for the family carer of a patient with ABI.^15,16,17^ The resultant depression, feelings of hopelessness and reduced quality of life experienced by family caregivers have been well documented.^17,18,19,20,21,22^ A recent study also found that many of the family members who care for a patient with ABI and who suffer from high levels of stress can have marked suicidal ideation.^14^

Furthermore, research has identified a significant correlation between stress and reduced coping.^9,23,24,25,26^ Although a degree of stress in life is inevitable and can be positive (referred to as eustress), prolonged negative stress as experienced by family members who care for a patient with ABI can reduce their ability to cope, which may negatively impact on the well-being of the patient with ABI. When considering the influence of chronic stress on the psychological and physiological well-being in these family members, it becomes evident that identifying ways of ameliorating its effect is imperative in order to help them to cope with the caregiver demands in the long-term. The use of a theoretical framework is elaborated on in more detail in the discussion section.

Despite these facts, there is a paucity of research in South Africa regarding the impact of stress on psychological and physiological health and coping skills of family members caring for a patient with ABI. Therefore, the objective of this study was to investigate the impact of stress on levels of depression, ill health and coping amongst family members and to highlight the profoundly negative impact of stress on the family caregivers’ health and their well-being, so that when healthcare professionals see these families in their practices, they will be more likely to enquire about the family caregivers’ experience of stress, and treat and refer as necessary. This study forms part of a larger investigation of caregiver burden or stress in family members who care for a patient with ABI.^14^

## Methodology

### Definitions of core terminology

For the purpose of this study, the following definitions apply.

‘Stress’ is defined as a person’s psychological, behavioural and physiological response to an event or set of circumstances that the individual interprets as being beyond his or her capacity to cope with, resulting in a sustained stress response to their stressor(s), and which can be measured, amongst others, through psychological screening.^25,27^

‘Acquired brain injury’ is a blanket term which is defined as neurological damage that occurs after birth either due to a traumatic event such as an injury to the head or a fall, motor vehicle collision or sports-related injury, or a non-traumatic incident, such as a brain tumour, meningitis, encephalitis, hypoxia and cerebrovascular accident (CVA).^1,28^

‘Coping’ is defined as a conscious effort towards solving problems and managing difficult emotions in order to master or minimise the impact of unhealthy stress. This involves developing coping strategies and skills which are adaptive and not maladaptive. Many different coping strategies have been identified. Typically, people use a mixture of these. From a psychological perspective, emotion-focused and problem-focused coping are, amongst others, important strategies.^25^ For our purposes, we have concentrated on problem-focused coping skills.

‘Depression’ incorporates the experience of feeling low, irritable and meaningless along with physiological and cognitive effects that profoundly undermine the person’s ability to cope with life.^29^ This includes Beck’s cognitive model (utilised in this study as mentioned in the discussion section) described the development of depression as comprising a triad of three fundamental components that include negative thought patterns about the self, environment and the future.^30^

‘Caregiver burden’ is defined as involving multiple factors and demands associated with being a caregiver, such as physical, emotional and socioeconomic adverse implications.^14,18^

### Study design

The research involved a cross-sectional descriptive study of family members who are caring for a patient with ABI. A mixed method design of data collection was used which included the administration of two standardised inventories to measure stress and depression, as well as self-report procedures (structured questionnaires and interviews).

### Sample

The research sample consisted of 80 family caregivers who are members of Headway Gauteng, located in Johannesburg, South Africa. Headway Gauteng is a registered non-profit organisation that offers a variety of support programmes to adults living with ABI, their families and caregivers. The study sample consisted of primary caregivers (72.5%) who are the foremost family members providing care for the patients with moderate to severe ABI, and secondary caregivers (27.5%) who are the family members who assist and support the primary caregivers. The patients with ABI that the family members care for, all attend Headway Gauteng where they were neuropsychologically screened. The age range at baseline of the sample of caregivers was 18 to 75 years (X^−^ = 49.6), with 75% (60) being females and 25% (20) being males.

### Data collection and analyses

The interviews (conducted between June 2018 and October 2019) with the family caregivers were in-depth and took approximately 2 hours to 3 hours each. They consisted of one-on-one discussion and included the research participants completing a questionnaire and two psychological measures as discussed later in the article. The principal author conducted the interviews individually at the Headway Hyde Park and Soweto branches in Johannesburg. The responses to the questionnaires were both verbal and written. Extensive notes were made and collated. For the purpose of this study, information gained from the following question was used as part of the questionnaire where the study participants were asked to answer yes or no to the question: ‘Do you believe that your current health status has been affected by your family member’s ABI? Yes/No, please elaborate’. Each participant gave further verbal and written information about how their caregiver burden was undermining their well-being and reducing their ability to cope. The research participants were also asked to write down the medical and psychological diagnoses that they have received since becoming a caregiver.

A stress symptom checklist (SSCL)^25,27,31^ was used to measure the research participants’ stress levels. The SSCL is a reliable, valid and clinically effective dichotomous-scaled 87-item checklist of the typical indicators and symptoms of negative stress.^27^ Rounded-off reliability coefficients range from 0.8 to 0.9, and validity (correlation) coefficients range from 0.5 to 0.6.^27^ Validity included content validity based on a discriminating item selection, criterion and construct validity, as well as convergent and discriminant validity. Items are categorised into three main subscales: physical symptoms (18 items), psychological symptoms (27 items) and behavioural symptoms (42 items). The highest total score is 87, with scoring categories being: low stress = 8 and below, mild stress = 9 to 15, moderate stress = 16 to 30, severe stress = 31 to 45 and profound stress = 46 and above.^27^ A total score of nine or higher across all three subscales indicates the onset of unhealthy stress for that research participant. The Beck Depression Inventory (BDI)^32^ was used to measure levels of depression. It is a 21-item multiple-choice self-report inventory which measures severity of depression. Individual scale items are scored on a 4-point continuum (0 = least, 3 = most), with a total score range of 0–63. We used the standard cut-off scores (0–9 = minimal depression, 10–18 = mild depression, 19–29 = moderate depression and 30–63 = severe depression).

The SSCL scores were non-normally distributed and, as a result, nonparametric statistical tests (including the Mann–Whitney) were used with a significance level of *p* = 0.05. Data were categorised on several dimensions. Scores obtained on the psychological and physiological subscales of the SSCL were calculated separately and the total scores were compared with the total scores obtained from the BDI in order to identify a relationship between elevated stress and depression that could have resulted in poor coping in the sample studied. As the focus was on the psychological and physiological stress symptoms, the behavioural subscale of the SSCL was excluded as its relevance was previously reported, and it reflected a significant correlation between high stress levels and behavioural stress symptoms and suicidal ideation in the participants.^14^ Interview and questionnaire responses combined with the scores on the SSCL and the BDI were analysed to determine whether the participants were coping and implementing effective coping skills. Research participants whose total SSCL stress scores fell in the moderate to severe categories, together with their BDI scores that indicated mild to severe depression taken with their self-reported physical and/or psychological diagnoses revealed that they were not coping with their caregiver burden. This was further supported by the qualitative analyses of the written information revealing their personal experience of high stress and caregiver burden which also indicated that they were not coping.

### Ethical consideration

Ethical approval for the study was obtained from the Biomedical Research and Ethics Committee, College of Health Sciences, University of KwaZulu-Natal, Durban, South Africa. Participants were provided with an information sheet which explained that their participation in the study was entirely voluntary and confidential. In addition, they understood that the information provided and the assessment procedures completed pertained specifically to their personal experience of caring for a relative with acquired brain injury.

## Results

**FIGURE 1:**
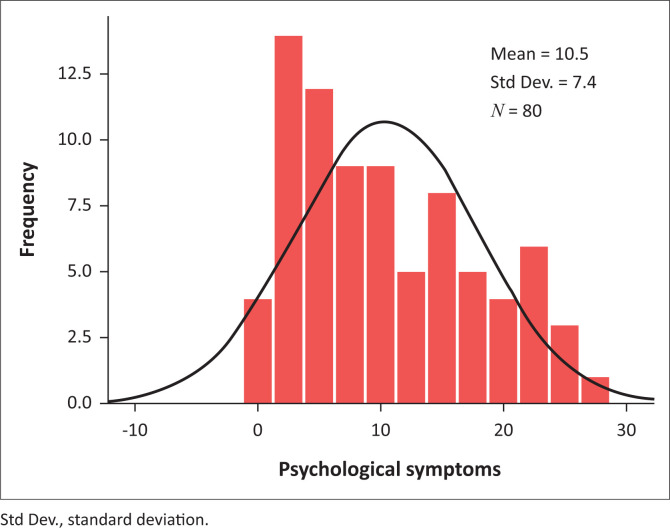
Psychological symptoms of stress.

**FIGURE 2:**
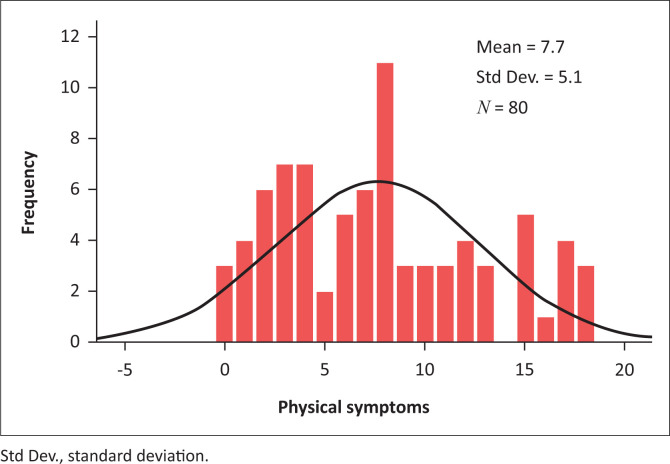
Physical symptoms of stress.

Results are reflected as a graphical representation in histograms of the psychological and physical symptoms recorded on the SSCL showing their non-normal distribution. From this, a significant psychological (Figure 1) and physical (Figure 2) health impact on the research participants can be seen. The scores on the psychological subcategory of the SSCL (Figure 1) were more elevated than the scores on the physical subcategory of the SSCL (Figure 2), although scores on both subcategories were indicative of inordinate stress and ill health-related consequences.

Self-report questionnaires and interview data (based on the qualitative results) corroborated the findings of the psychological and physical stress symptom scores on the SSCL. Overall, 62.5% (50) of the sample had received a medical and/or psychological diagnosis after they became a caregiver. Qualitative analyses of the participants’ questionnaire responses revealed that they reported marked variability in health effects. For example, some (18.75%) of the participants developed only physical ill health and there was also variability in this, such as hypertension, cancer, stroke and autoimmune conditions. Others (22.5%) developed only psychological disorders, such as depression and anxiety, whereas a significant percentage (21.25%) developed both physical and psychological disorders. The verbal and written feedback from the research participants included insightful information about how they believed that caregiver stress was impacting their health. For example, a young woman caring for her sister said the following:

‘Since my sister’s ABI, I have had to seek psychiatric treatment for my stress disorder and I am now taking a handful of medications everyday just to cope, but before her injury, I occasionally took a headache tablet and nothing else and this sums up how my life has changed.’ (Female, sister, caregiver)

A husband caring for his wife reported:

‘I have been ignoring my own health since my wife’s ABI and recently I was diagnosed with spinal ependymoma, which I believe is directly due to the extreme strain of the past few years.’ (Male, husband, caregiver)

A mother caring for her daughter reported:

‘I can’t cope without my medication for depression and anxiety’ (Female, caregiver) and ‘since being the primary caregiver my previously controlled hypertension has now become uncontrollable despite taking the medication’ (Female, mother, caregiver).

**FIGURE 3:**
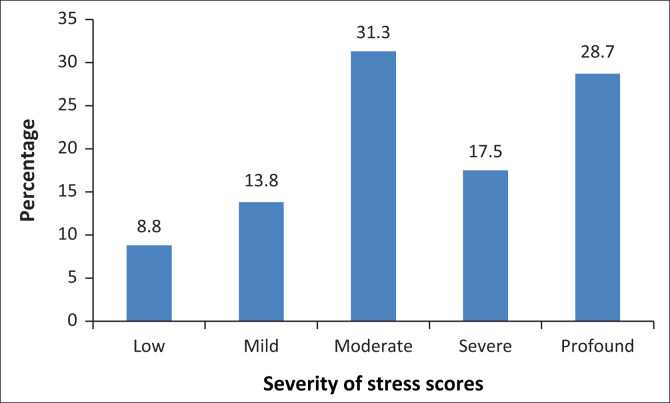
Total stress symptom checklist scores (*N* = 80).

All these research participants reported that their health-related conditions began after the onset of their inordinate stress associated with being a caregiver of a patient with ABI. Additionally, total scores on the SSCL and BDI indicated elevated levels of both stress and depression respectively. As can be seen in Figure 3, 77.5% (62) of the research participants’ scores fell in either the moderate, severe or profound stress categories.

**FIGURE 4:**
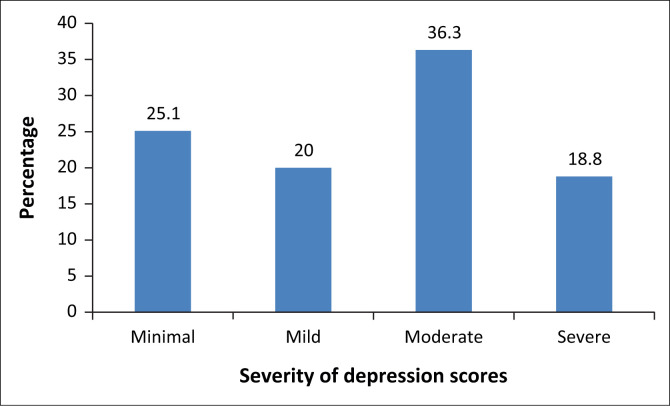
Total Beck Depression Inventory scores (*N* = 80).

When compared with the elevated stress scores, 75% (60) of the sample were also found to have either mild, moderate or severe depression as measured on the BDI (Figure 4), confirming a positive link between stress, depression and poor coping skills in the sample studied.

Taking all of this information into consideration, the research participants were determined to be either coping or not coping. From the total sample, 67.5% (54) were found to be not coping and 32.5% (26) were found to be coping, but these participants also had areas of concern with respect to their health risks and future well-being.

## Discussion

The results of this study (both qualitative and quantitative) highlighted the psychological and physiological toll on family members caring for a patient with ABI and the difficulty in coping with their caregiver demands. These findings were identified despite the research participants all having substantial support from well-trained therapists at Headway Gauteng. However, in our experience many caregivers do not receive or have access to such support. On a societal level, there are likely many families experiencing serious physiological and psychological negative health effects as a result of the ongoing stress of caring for a patient with ABI. This increases the risk of additional strain on limited access to resources for patients with ABI especially when having to deal with many related diverse problems including psychiatric disorders and the underestimation of stress-related conditions, such as post-traumatic stress disorder.^2,14,33,34^

The theoretical framework underpinning this research is based, in part, on an integrated biopsychosocial (BPS) model of health described by Schlebusch.^9,24,25,27^ This model has at its basis the general systems theory,^35^ which contributed to the refinement of the medical model of health and involved a movement away from a reductionistic view of health to a more holistic perspective.^36^ The BPS model emphasises that both health and ill health involve an interaction between biological, psychological and social factors as, for example incorporated in the SSCL.^9,24,25,27,35,36^ This approach has been particularly useful in appreciating the negative impact of acute and chronic stress on physiological and psychological well-being in this study’s participants. The high percentage of psychological and physical stress-related symptoms, in addition to the total stress scores associated with depression and ill health as found in the research participants, highlighted the systemic impact of stress on them.

People’s predispositions to the development of physical and/or psychological disorders are understood to be highly variable,^9,25,27,37,38,39^ as also found in this study. When coping skills are adaptive and allostasis occurs, the individual under stress should be better placed to maintain good health. However, pathophysiology can occur when stress reduces coping skills which may become maladaptive, causing disruptions to the process of allostasis and resulting in AL.^8^ This helped improve our understanding of why this study’s participants reported high levels of ill health. Because of this, the BPS model was further complemented by the utilisation of the cognitive model in terms of the psychological impact of stress^24,40^ which describes individuals who are not coping with stress as being unable to process or rationalise the stressor(s). The effect is distress along with a feeling of being unable to cope, influence or control the stressor(s).^25^ As part of this, the psychological stress response begins with the individual’s perception and interpretation of their stressor(s). Many of this study’s participants, who had a diagnosis of depression and/or ill health, reported feeling overwhelmed by and trapped in their role as caregiver. This feeling of helplessness was described by many of the research participants during interviews. It has the potential to produce a markedly dysfunctional psychological state in a person who was previously healthy. The small number of family caregivers who had minimal signs of stress, depression or ill health before or after becoming a caregiver all described finding meaning in the caregiver experience which helped them to develop more adaptive coping skills. For example, a mother caring for her daughter said that her daughter’s ABI had ‘brought her back to her’ as before the injury their relationship had been distant. Another carer described finding great joy in being able to comfort and alleviate the suffering of her partner with ABI. In addition, some of the family members who were coping believed that their caregiver duties had a spiritual significance for themselves and the patient. They had developed effective coping responses which acted as mitigating or interceding factors between stressors and their stress response.

If coping skills remain maladaptive, the risk for psychological and physiological health complications may increase significantly as borne out in the case of most of our research participants. These findings have implications for the family members’ ability to cope over time with the demands of being a caregiver. There is a need for appropriate support and education for people providing care to patients with ABI. Family members would benefit from education regarding their stress response and how it can affect them. Effective assessment and stress prevention require a comprehensive approach in order to identify the extent to which these factors negatively interact and place family members who care for patients with ABI at higher risk for problematic health outcomes. Our findings underscore the importance of medical and mental health professionals working together in order to prevent and/or manage the negative physical and psychological health effects of inordinate stress in the sample that we studied and which may be applicable to other high-risk family members caring for patients with debilitating conditions. When considering the influence of chronic stress on the psychological and physiological well-being in these family members, it becomes evident that identifying ways of ameliorating its effect is imperative in order to help them to cope with the caregiver demands in the long term. In this regard, protective factors such as developing effective coping strategies can help to mitigate the negative effects of inordinate stress and potentially contribute to improved resilience, adaptation and coping.

The limitations of this study include the fact that the research participants were all drawn from a single organisation, Headway Gauteng, which operates in Johannesburg, Hyde Park and Soweto. The study participants all have access to psychological support services which implies that the findings may be different in a population of family caregivers who do not have access to such support. In addition, the study consisted of a small sample size (*N* = 80) and therefore the findings cannot necessarily be generalised.

## Conclusion

The relationship between elevated stress, depression, ill health and reduced coping skills in family members who care for patients with ABI has, in the past, not been extensively researched, especially in developing countries including South Africa. The high percentage of stress, depression and ill health amongst our sample suggests that they are not coping as well as they could with their self-perceived burden of being a family caregiver of a patient with ABI. Given the above, the conditional probability is high that if stress experienced by these family members is not adequately managed, it may exacerbate any physical and psychological health complications. This provides compelling evidence of the value of psychological screening for elevated stress and poor coping in family members caring for a patient with ABI. In this regard, we recommend a collaborative effort between medical and psychological health practitioners which could help to ensure a more holistic and inclusive approach to treatment procedures and interventions to improve coping skills in these individuals.
